# Novel Approaches for Systems Biology of Metabolism-Oriented Pathogen-Human Interactions: A Mini-Review

**DOI:** 10.3389/fcimb.2020.00052

**Published:** 2020-02-13

**Authors:** Tunahan Çakır, Gianni Panagiotou, Reaz Uddin, Saliha Durmuş

**Affiliations:** ^1^Department of Bioengineering, Gebze Technical University, Kocaeli, Turkey; ^2^Leibniz Institute for Natural Product Research and Infection Biology, Hans Knoll Institute, Jena, Germany; ^3^Dr. Panjwani Center for Molecular Medicine and Drug Research, International Center for Chemical and Biological Sciences, University of Karachi, Karachi, Pakistan

**Keywords:** infectious diseases, genome-scale metabolic networks, pathogen-host interactions, transcriptome, metabolome, gut microbiota, dual omics

## Abstract

Pathogenic microorganisms exploit host metabolism for sustained survival by rewiring its metabolic interactions. Therefore, several metabolic changes are induced in both pathogen and host cells in the course of infection. A systems-based approach to elucidate those changes includes the integrative use of genome-scale metabolic networks and molecular omics data, with the overall goal of better characterizing infection mechanisms for novel treatment strategies. This review focuses on novel aspects of metabolism-oriented systems-based investigation of pathogen-human interactions. The reviewed approaches are the generation of dual-omics data for the characterization of metabolic signatures of pathogen-host interactions, the reconstruction of pathogen-host integrated genome-scale metabolic networks, which has a high potential to be applied to pathogen-gut microbiota interactions, and the structure-based analysis of enzymes playing role in those interactions. The integrative use of those approaches will pave the way for the identification of novel biomarkers and drug targets for the prediction and prevention of infectious diseases.

## Introduction

One major focus of systems biology in medical research is the integrated use of high-throughput omics data and molecular interaction networks, with the overall goal of elucidating molecular mechanisms of diseases to identify potential biomarkers and personalized treatment strategies. A wealth of research has been conducted to apply this approach on infectious diseases by focusing on cell metabolism. This included collecting transcriptomic, proteomic, or metabolomic data from pathogens or infected hosts on one side, and developing interaction models focusing on the enzymes and reactions of crucial metabolic pathways on the other side (Bumann, 2009).

Rather than investigating host and pathogen metabolisms separately, novel systems biology based approaches consider the pathogen-host interaction (PHI) system as a whole to elucidate the mechanisms of infection. Here, omics data is collected from both organisms in a single experiment, termed dual-omics data, focusing on all the molecules within the PHI system (Westermann et al., 2017). Partly possible thanks to the developments in Next Generation Sequencing technologies, this enables the generation of valuable omics data in shorter times.

Reconstruction of microorganism-specific metabolic networks based on genome sequences is a powerful approach when combined with the computational tools that enable the prediction of phenotypic indicators such as rates of metabolic reactions, activities of metabolic pathways, effect of gene deletion on growth. When applied to pathogenic organisms, it is possible to identify potential biomarkers and drug targets with this approach (Kim et al., 2012; Dunphy and Papin, 2018). There are several studies that report the reconstruction of genome-scale metabolic networks (GMNs) for pathogens, which are reviewed elsewhere (Cesur et al., 2018). The generation of dual-omics data makes it possible to extend the genome-scale metabolic network modeling of pathogens to PHI systems, by simultaneously considering pathogen and host metabolisms.

One crucial contribution of PHI-based integrated metabolic approach is to identify host non-homologous metabolic pathways of pathogenic resistant microorganisms to propose new drug targets. Resistant pathogens use their unique metabolic pathways to overcome the harsh conditions produced by the administered antibiotics. Identification of those pathways and related enzymes using protein homology and structure is important to predict novel drug targets for infectious diseases.

**Figure 1:**
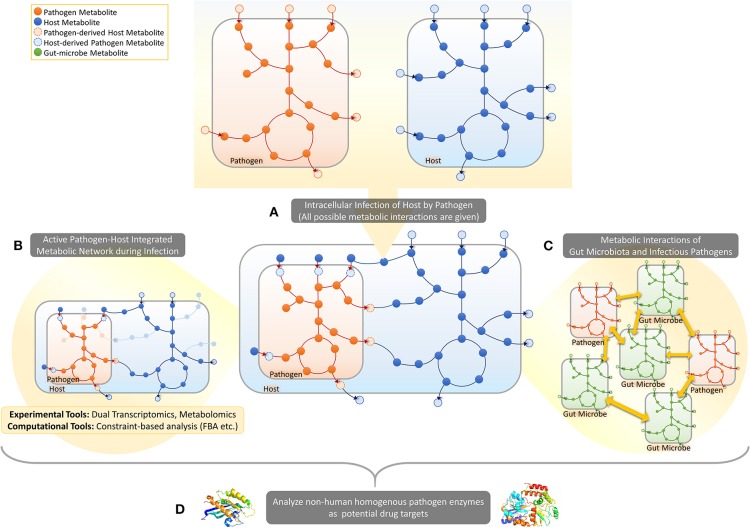
Pathogen-host metabolic interactions. **(A)** When a host cell is intracellularly infected by a pathogen, several metabolite exchanges are possible between the pathogen and the host. **(B)** In response to infection several metabolic pathways will get activated/inactivated in both pathogen and host in addition to the activation of some metabolic exchanges between the two cells. Dual-omics studies provide experimental tools to take a snapshot of the PHI systems, enabling prediction of active/inactive metabolic reactions in a specific infection time/condition. Constraint-based analysis approaches, on the other hand, provide computational tools to map omic data on integrated pathogen-host metabolic networks to identify active parts. **(C)** Such metabolic-network based frameworks can also be developed to predict metabolic interactions between pathogens and gut microbes in gut. **(D)** The integrated metabolic networks can be processed for the prediction of selective drug targets and corresponding drugs.

This mini-review focuses on the recently emerging PHI-based approach to metabolism in human infection, covering studies using dual-omics approach, pathogen-host integrated GMNs and structure and homology based analysis of enzymes. The reviewed approaches are illustrated in Figure 1.

## Metabolism-Oriented Dual Omics of Pathogen-Host Interactions

It is possible to perform simultaneous omics analysis of the pathogen and host together, generating dual omics data focusing on both organisms (Durmuş et al., 2015; Westermann et al., 2017). First examples of metabolism-oriented analysis of dual transcriptomic data have recently been available, which aim to gain insights specifically on the metabolism of the pathogen and the host during infection. These examples are reviewed below.

Zimmermann et al. (2017) collected metabolomics and dual RNA-Seq data of the macrophages infected by *Mycobacterium tuberculosis*. Integrating the omics data with the combined genome-scale metabolic network model of macrophage and *M. tuberculosis* provided insights on the metabolic robustness and resistance of the bacteria to metabolic interventions. Calculation of metabolic fluxes using the combined GMN constrained by the dual RNA-Seq data generated predictions of co-utilization of 33 different carbon sources. The results enlightened the substrates directly used by the pathogen as biomass precursors and the ones further metabolized for energy or building blocks. On the other hand, pathogen/host joint metabolomics and dual transcriptomics data were investigated together to shed light on the metabolic changes during *Toxoplasma gondii* infection of human (Olson et al., 2018). Paired analysis of joint metabolome and dual transcriptome data uncovered the manipulation of the host metabolome by *Toxoplasma* and identified sedoheptulose biphosphatase driven ribose synthesis from glucose as a novel metabolic capability of the parasite. The identified metabolic enzyme was proposed as a potential drug target since it was not present in human. In another study, Tucey et al. (2018) investigated the crosstalk between glucose metabolism of immune cells and that of pathogenic fungus *Candida albicans*, focusing on differentially expressed pathogen and host genes during infection. From the analysis of dual RNA-seq data, the glucose competition by *C. albicans* was found to be responsible for the death of lots of infected macrophages. The results provided evidence for a key role of host glucose homeostasis *in vivo* during animal infection, and it was proposed that a glucose-rich diet improved host outcomes in *Candida* infection. In a recent study, ulcer-associated pathogen *Haemophilus ducreyi* was investigated in detail by collecting dual transcriptomic and host metabolomic data from infected human tissue (Griesenauer et al., 2019). The results suggest the consumption of ascorbic acid and adaptation of anaerobic metabolism as survival mechanisms by the pathogen in glucose-poor abscess environment.

There are a number of other dual-transcriptomic analyses of PHI systems in literature without a specific focus on metabolism, but they also briefly report associated metabolic alterations. These studies are reviewed in Table 1.

**Table 1:** Key metabolic findings from dual transcriptome analysis of pathogen-mammalian host systems, given in chronological order.

**References**	**Pathogen-mammalian host system**	**Key metabolic findings in response to infection**
Humphrys et al. (2013)	*Chlamydia trachomatis*—Human epithelial cell monolayers	- Early up-regulation of many transferases and transporters in the pathogen, hinting at translocation of host cell metabolites - Up-regulation of riboflavin biosynthesis enzymes as a possible strategy of the pathogen for early iron acquisition
Pittman et al. (2014)	*Toxoplasma gondii*—Mouse brain	- Up-regulation of metabolic processes in the pathogen and down-regulation of metabolic processes in the host only in chronic infection (not observed in acute infection)
Baddal et al. (2015)	*Haemophilus influenzae*—Human ciliated bronchial epithelial cells	- Down-regulation of central metabolism and biosynthesis pathways in the pathogen together with up-regulation of transporters, indicating the effect of host substrates
Damron et al. (2016)	*Pseudomonas aeruginosa*—Mouse lung	- Up-regulation of gluconeogenesis, polysaccharide biosynthesis and arginine metabolisms in the pathogen - Up-regulation of heme acquisition in the pathogen and up-regulation of iron sequestration systems in the host, hinting at the competition between the pathogen and the host for iron
Fernandes et al. (2016)	*Leishmenia major* and *Leishmenia amazonensis*—Human macrophages	- Down-regulation of valine, leucine, isoleucine, lysine degradation, and fatty acid biosynthesis pathways in *L. major*
Li et al. (2017)	*Actinobacillus pleuropneumonia*—Mouse lung	- Up-regulation of central carbon, amino acid, fructose/mannose and inorganic iron metabolisms in the pathogen-Up-regulation of anaerobic in the pathogen, indicating an anaerobic host environment
Nuss et al. (2017)	*Yersinia pseudotuberculosis*—Mouse Peyer's patches	- Up-regulation of carbon uptake (glucose, mannose, fructose, glucuronate) systems, and down-regulation of TCA cycle, fatty acid oxidation and respiratory chain in the pathogen, suggesting a switch to fermentative metabolism - Down-regulation of metabolic enzymes and glucose transporters in the host, pointing to shutdown of pivotal functions
Niemiec et al. (2017)	*Candida albicans*—Human neutrophils	- Up-regulation of acetate and carboxylic acid catabolisms, and down-regulation of glycolysis, energy reserve and monocarboxylic acid metabolisms in the pathogen, which are mostly regulated by only four transcription factors
Petrucelli et al. (2018)	*Trichophyton rubrum*—Human keratinocytes	- Up-regulation of glyoxylate cycle genes, hinting at metabolic flexibility, and a carboxylic acid transporter gene, hinting at improved nutrient assimilation, in the pathogen
Kiedrowski et al. (2018)	*Staphylococcus aureus*—Cystic fibrosis airway epithelial cells	- Up-regulation of aminoacid and lipid metabolisms in pathogen during coinfection with respiratory syncytial virus - Up-regulation of amino acid catabolism genes in the pathogen in the coinfection, suggesting utilization of host-secreted proteins by the pathogen
Jacquet et al. (2019)	*Staphylococcus aureus*—Mouse skin	- Down-regulation of amino acid transport and metabolism and up-regulation of glycolysis and TCA cycle in the pathogen from diabetic infected mice compared to the pathogen from infected control - Down-regulation of lipid, vitamin, and mineral metabolism in the host in response to infection
Muñoz et al. (2019)	*Candida albicans*—Mouse macrophages	- Up-regulation of glucose/carbohydrate transport, glyoxylate metabolism and fatty acid catabolism in the pathogen - Down-regulation of glyoxylate metabolism and up-regulation of glycolysis, gluconeogenesis, and fatty acid biosynthesis in the host
Minhas et al. (2019)	*Streptococcus pneumoniae*—Mouse lung	- Alterations in multiple sugar transporters and carbohydrate metabolism in the pathogen due to a SNP in rafR gene of the pathogen

*The findings are mostly based on the enrichment analysis of differentially expressed genes. For most of the systems in the table, only the findings for the pathogen side are given since the host side is reported to alter mainly immune signaling pathways rather than metabolic pathways*.

## Genome-Scale Metabolic Networks of Pathogen-Host Interactions

Since pathogens tightly interact with the host cells during infection, they cause alterations in the level of available metabolites in the host cell, leading to rewiring of host metabolic pathways. Therefore, focusing solely on pathogen metabolism in infection research has limitations. The genome-scale reconstruction of pathogen-host integrated metabolic networks is therefore crucial for a holistic analysis of metabolic changes in infection. Guidelines specific to the reconstruction of such integrated metabolic networks were published (Jamshidi and Raghunathan, 2015; Raghunathan and Jamshidi, 2018). Contrary to its utmost importance, studies with such integrated metabolic networks are currently limited in number in literature. The details of the available studies are reviewed in this section.

The first integrated genome-scale metabolic network of pathogen-host interactions was reported for the *Plasmodium falciparum* infection in erythrocytes, where infection-specific gene expression data of the parasite was incorporated into flux prediction algorithm (Huthmacher et al., 2010). The authors reconstructed a metabolic network of human erythrocytes with 349 reactions and a network of 998 reactions controlled by 579 genes for the malaria pathogen. In the integrated network simulations, *P. falciparum* was forced to consume some host metabolites to better represent infection characteristics. Compared to the use of only the metabolic network of the pathogen, the simulation of the integrated metabolic network better predicted the metabolites exchanged between pathogen and the host when integrated with transcriptomic data. The authors later applied the same approach for the hepatocyte infection of *P. falciparum*, the first site of infection in human body for this pathogen (Bazzani et al., 2012). The GMN of human hepatocyte, Hepatocyte1, includes 2,539 reactions controlled by 704 genes. The network was integrated with an updated network of *P. falciparum* with 1,394 reactions and 579 genes. By leaving all pathogen-host metabolite exchange rates unconstrained, they performed gene deletion and reduced fitness simulations. The integrated analysis enabled the prediction of 24 enzymes as selective drug targets, which are essential in the pathogen but non-essential in hepatocytes.

Another early example of integrated metabolic network approach is for the pathogen *M. tuberculosis* and its infection of alveolar macrophages (Bordbar et al., 2010). To this aim, the authors used a genome scale reconstruction of the pathogen with 661 genes and 1,027 reactions. The host metabolic network was derived from the generic human metabolic reconstruction Recon1 with the help of macrophage-specific gene expression data, leading to 3,394 reactions and 1,410 genes. This corresponds to the removal of 86 genes from the Recon1 model. The exchange of metabolites between the two networks was allowed based on literature information. The authors also updated the composition of biomass reaction in the pathogen network to better represent *in vivo* infection conditions. They showed that gene deletion simulations with the integrated network leads to improved gene essentiality predictions when compared to the use of the pathogen metabolic network alone. The pathogen-host metabolic network was also integrated with the transcriptome data from infected macrophages. This shed light on the effect of three different types of *M. tuberculosis* infection on the metabolisms of the pathogen and the host. Later, the same integrated metabolic network was integrated with dual RNA-seq data, as mentioned in the previous section (Zimmermann et al., 2017). The results predicted a multi-nutrient strategy for the pathogen during early infection. Additional gene essentiality simulations indicated that the number of essential genes decreases during infection. Recently, others investigated the same pathogenic infection of macrophages by using improved metabolic networks (Rienksma et al., 2019). The *M. tuberculosis* metabolic network used included 1,192 reactions and 915 genes. As the host GMN, a more recent reconstruction of generic human metabolism, Recon 2.2, was used, with 7,785 reactions and 1,675 associated genes. Dual RNA-seq data from macrophage-like THP-1 cells infected with a close relative, *M. bovis* BCG, was integrated with the reconstructed pathogen-host metabolic network. First, condition-specific biomass reactions were created by the help of transcriptome data for both cell types. Later, the response of host and pathogen metabolic networks to 11 metabolically active anti-tuberculosis drugs was individually simulated by gradually increasing the flux through the affected reactions. This enabled the identification of reroutings in the pathogen metabolism.

## Genome-Scale Metabolic Networks for the Interaction of Gut Microbiota and Infectious Pathogens

Microbial ecology has witnessed tremendous progress over the last decade empowered by new sequencing technologies. These innovations in DNA sequencing resulted in a paradigm shift concerning our understanding of pathogenic processes and gave birth to a new scientific concept, namely the “pathobiome” (Vayssier-Taussat et al., 2014). In the pathobiome concept, the microbial community containing a pathogenic agent exerts a major influence on the persistence, transmission, and evolution of the pathogen. For example, reduction of indigenous Lachnospiraceae in the human gut following antibiotic treatment creates a niche for *Clostridium difficile* infections (Fleming-Davies et al., 2017; Jenior et al., 2017). Sexually transmitted infections such as *Chlamydia trachomatis* and human papilloma virus have been associated with reduction of *Lactobacillus* species that results in high-diversity vaginal microbiota (Sewankambo et al., 1997). Interestingly, metabolic products or functions of the microbiota can be critical for the survival of invading species. The microbiota regulates the bile acid pool whereas specific bile acids are required for the maximum germination of *C. difficile*. Short chain fatty acids levels, which can limit the growth and colonization of pathogens by disrupting the intracellular pH homeostasis, as in the case of *Salmonella enterica*, are also influenced by the composition of the microbiota (McHan and Shotts, 1993; Horswill et al., 2001; Jacobson et al., 2018). It is therefore mandatory to identify those species that contribute, either as partners or antagonists, to pathogen's survival, extinction or dispersal by applying concepts and approaches of systems biology. Developing systems biology frameworks would allow us to go beyond the identification of mere statistical co-occurrence patterns and instead develop computational models that describe the spatio-temporal dynamics of the pathogen and the functional interactions with its biotic environment.

GMNs have only recently been used for gaining a mechanistic insight into the interactions between communities and their host, as well as, between ecosystem members. For the former, one of the first attempts was based on the “supra-organism” concept. By defining the metagenome metabolic network of 124 healthy, obese and with inflammatory bowel disease individuals, Jacobsen et al. (2013), could reveal clusters of high, medium and low metabolic potential. The abundance of specific genera was shown to be a key factor for the metabolic potential of the gut community. Recently, Garza et al. (2018) combined GMNs of individual species to predict the metabolic status of the whole community. Using an optimization framework that takes as input metagenomic species abundance levels and their GMNs, the authors could produce meaningful predictions for the metabolic environment. This approach provides a starting point for assessing the risk of a microbial infection based on the full composition of the environment. Recent pioneering studies providing a large number of high-quality GMNs for gut bacteria enable *in silico* studies of gut metabolic function and interaction (Magnúsdóttir et al., 2017; Machado et al., 2018). These resources have exemplified the study of gut microbes and their respective pairwise interactions but have not been used so far to study the interaction with pathogenic species. From these >1,000 human gut bacteria with GMNs available, we could create a “Potential Therapeutic Microbial Pool (PTMP)” that consists of those microbes that (i) are not pathogens or previously associated with diseases, and (ii) are more stable against perturbations in healthy individuals. Subsequently, and based on computed pairwise growth interactions with gut pathogens, such as *C. difficile, E. coli, Enteroccocus* spp., and *Campylobacter* spp., among others, we can identify microbes from the aforementioned PTMP that have a competitive relationship with the pathogen. Such computational frameworks could enable the design of microbiome-based prophylactic and therapeutic interventions for vulnerable-to-infections individuals.

## Structure-Based Approaches to Analyze Enzymes in Pathogen-Host Interactions

PHI-based integrated metabolic approach can be applied to discover enzymes as potential drug targets. Computational subtractive genomics is one such approach to shortlist or prioritize enzymes in unique metabolic pathways of infectious pathogens as drug targets. The enzymes are filtered based on the non-host protein homology as well as essentiality for the survival of the pathogen. The structure-based methods such as homology modeling, molecular docking and molecular dynamics simulations are used to classify the candidate enzymes in terms of structure and function, as reviewed below.

Hossain et al. (2018) presented a very interesting application of the subtractive genomics method on a bacterial species *Campylobacter jejuni* RM1221 (*CjR*). The bacteria *CjR* is now resistant to many antibiotics. Therefore, there is a need to predict new drug targets based on unique and new pathways of the resistant *CjR*. In the first phase, the authors used the complete protein set of the resistant *CjR* to prioritize the essential proteins of *CjR* taking role in unique metabolic pathways. These unique pathways were pathogen specific only, therefore, absent in the host organism. In the next step, the authors filtered out the human homolog proteins. Finally, using this subtractive genomics strategy, they identified 38 eligible therapeutic targets, about 10 of which were metabolic enzymes. They studied corresponding 3D structural interactions with FDA-approved drugs. Additionally, they identified peptidoglycan biosynthesis as a pathway based drug target, to be explored by experimental methods. To help future research, a comprehensive database was also made, and it has 3D structure and other related data of the listed drug targets.

Sulfur metabolism pathway is one of the essential pathways of microorganisms to survive as a counter strategy to overcome the oxidative defense of a human host during the long latent phase of their life cycle, particularly, in anaerobic pathogens such as *Entamoeba histolytica, Leishmania donovani, Trichomonas vaginalis*, and *Salmonella typhimurium*. Since the Sulfur metabolism pathway is absent in human and all other mammals, the enzymes taking role in this pathway were best considered as drug targets and subjected to the structure based methods to predict potential strong binders (Mazumder and Gourinath, 2016). The Sulfur metabolism in microorganisms is through the cysteine biosynthetic pathway, which helps the microorganisms in their survival and pathogenicity. Cysteine biosynthesis pathway involves two major enzymes and these are: (i) serine acetyl transferase (SAT) and (ii) O-acetylserine sulfhydrylase (OASS). Among these two enzymes, the availability of the crystal structure of OASS made it best target to focus for the structure based computational studies. In addition to its absence in human host, the OASS is highly conserved between above-mentioned pathogens. In the study of Mazumder and Gourinath (2016) the authors have shown that the intermediate state of the enzyme could be considered as the best option to design inhibitor against OASS instead of closed and open conformation of this enzyme.

The pathogens and host relationship is governed by the Protein-Protein Interactions (PPIs) and several of those interactions can involve enzymes. With methicillin resistant *Staphylococcus aureus* (MRSA), which is the main source of nosocomial infection worldwide, Uddin et al. (2017) developed a network of PPI by using the Interolog method. The interolog method is based on the hypothesis that if two proteins interact in one species then there is a likely chance that a set of the ortholog of interacting proteins will also interact in the cross species. Hence, in the study of Uddin et al. (2017) the pathogen-host PPIs between MRSA and Humans were predicted as possible drug targets by considering various filters. A distant homolog approach was employed to find the MRSA homologs in human host using PSI-BLAST. The Database of Interacting Protein (DIP) was also used to find the direct homologs of the pathogen proteins in human host. As a result, the common pathogen-host PPIs were predicted and out of which the most repeated entry i.e., MRSA Histone Deactylase (HDAC) was proposed as a new drug target, against which one can propose new chemical compounds as potential inhibitors. The structure of the HDAC enzyme was modeled using the SWISS-MODEL and a small-scale chemical compounds library was docked on the binding site of the MRSA HDAC. This computational screening resulted in compounds that showed theoretical strong binding with the HDAC according to their estimated energetics. The predicted strong binders were proposed as potential inhibitors against MRSA HDAC.

## Conclusion

With the birth of dual-omics technologies, we have entered a new era where interactions between pathogens and their host can be directly predicted to unravel molecular mechanisms of infectious diseases. Although integrated pathogen-host GMNs are currently scarce, we believe that the increase in the number of dual omics datasets, especially dual RNA-seq studies, will pave the way for the reconstruction of more and more integrated GMNs (Figure 1). Mapping dual-omics data from different states of infection on those models will lead to infection-specific prediction of metabolic phenotypes. Taking into account the crucial role of pathobiomes in infection, the balance between pathogens and microbiota in human gut is another dimension to analyze infectious diseases by using GMNs. We will witness reconstruction of integrated microbiota-pathogen GMNs or even multi models covering microbiota-pathogen-host GMNs to enable deeper interpretation of metagenomics datasets. The combined use of here-reviewed approaches for metabolism-oriented PHIs will certainly contribute positively to the drug research via identifying novel candidate enzymes as targets.

## Author Contributions

TÇ conceived the study. TÇ, GP, RU, and SD wrote the manuscript.

### Conflict of Interest

The authors declare that the research was conducted in the absence of any commercial or financial relationships that could be construed as a potential conflict of interest.

## References

[B1] Baddal B. Muzzi A. Censini S. Calogero R. A. Torricelli G. Guidotti S.. (2015). Dual RNA-seq of nontypeable *Haemophilus influenzae* and host cell transcriptomes reveals novel insights into host-pathogen cross talk. mBio 6, e01765–e01715. 10.1128/mBio.01765-15 26578681 PMC4659474

[B2] Bazzani S. Hoppe A. Holzhütter H. G. (2012). Network-based assessment of the selectivity of metabolic drug targets in *Plasmodium falciparum* with respect to human liver metabolism. BMC Syst. Biol. 6:118. 10.1186/1752-0509-6-118 22937810 PMC3543272

[B3] Bordbar A. Lewis N. E. Schellenberger J. Palsson B. Ø. Jamshidi N. (2010). Insight into human alveolar macrophage and *M. tuberculosis* interactions via metabolic reconstructions. Mol. Syst. Biol. 6:422. 10.1038/msb.2010.68 20959820 PMC2990636

[B4] Bumann D. (2009). System-level analysis of Salmonella metabolism during infection. Curr. Opin. Microbiol. 12, 559–567. 10.1016/j.mib.2009.08.004 19744878

[B5] Cesur M. F. Abdik E. Güven-Gülhan Ü. Durmuş S. Çakir T. (2018). Computational systems biology of metabolism in infection in Metabolic Interaction in Infection, Experientia Supplementum, vol. 109, eds Silvestre R. Torrado E. (Cham: Springer), 235–282. 10.1007/978-3-319-74932-7_6 30535602

[B6] Damron F. H. Oglesby-Sherrouse A. G. Wilks A. Barbier M. (2016). Dual-seq transcriptomics reveals the battle for iron during *Pseudomonas aeruginosa* acute murine pneumonia. Sci. Rep. 6:39172. 10.1038/srep39172 27982111 PMC5159919

[B7] Dunphy L. J. Papin J. A. (2018). Biomedical applications of genome-scale metabolic network reconstructions of human pathogens. Curr. Opin. Biotechnol. 51, 70–79. 10.1016/j.copbio.2017.11.014 29223465 PMC5991985

[B8] Durmuş S. Çakir T. Özgür A. Guthke R. (2015). A review on computational systems biology of pathogen–host interactions. Front. Microbiol. 6:235. 10.3389/fmicb.2015.00235 25914674 PMC4391036

[B9] Fernandes M. C. Dillon L. A. Belew A. T. Bravo H. C. Mosser D. M. El-Sayed N. M. (2016). Dual transcriptome profiling of *Leishmania*-infected human macrophages reveals distinct reprogramming signatures. mBio 7, e00027–e00016. 10.1128/mBio.00027-16 27165796 PMC4959658

[B10] Fleming-Davies A. Jabbar S. Robertson S. L. Asih T. S. N. Lanzas C. Lenhart S. (2017). Mathematical modeling of the effects of nutrient competition and bile acid metabolism by the gut microbiota on colonization resistance against *Clostridium difficile* in Women in Mathematical Biology, eds Layton A. Miller L. (New York, NY: Springer), 137–161. 10.1007/978-3-319-60304-9_8

[B11] Garza D. R. van Verk M. C. Huynen M. A. Dutilh B. E. (2018). Towards predicting the environmental metabolome from metagenomics with a mechanistic model. Nat. Microbiol. 3, 456–460. 10.1038/s41564-018-0124-8 29531366

[B12] Griesenauer B. Tran T. M. Fortney K. R. Janowicz D. M. Johnson P. Gao H.. (2019). Determination of an interaction network between an extracellular bacterial pathogen and the human host. mBio 10, e01193–e01119. 10.1128/mBio.01193-19 31213562 PMC6581864

[B13] Horswill A. R. Dudding A. R. Escalante-Semerena J. C. (2001). Studies of propionate toxicity in *Salmonella enterica* identify 2-methylcitrate as a potent inhibitor of cell growth. J. Biol. Chem. 276, 19094–19101. 10.1074/jbc.M100244200 11376009

[B14] Hossain M. U. Omar T. M. Alam I. Das K. C. Mohiuddin A. K. M. Keya C. A.. (2018). Pathway based therapeutic targets identification and development of an interactive database CampyNIBase of *Campylobacter jejuni* RM1221 through non-redundant protein dataset. PLoS ONE 13:e0198170. 10.1371/journal.pone.0198170 29883471 PMC5993290

[B15] Humphrys M. S. Creasy T. Sun Y. Shetty A. C. Chibucos M. C. Drabek E. F.. (2013). Simultaneous transcriptional profiling of bacteria and their host cells. PLoS ONE 8:e80597. 10.1371/journal.pone.0080597 24324615 PMC3851178

[B16] Huthmacher C. Hoppe A. Bulik S. Holzhütter H. G. (2010). Antimalarial drug targets in *Plasmodium falciparum* predicted by stage-specific metabolic network analysis. BMC Syst. Biol. 4:120. 10.1186/1752-0509-4-120 20807400 PMC2941759

[B17] Jacobsen U. P. Nielsen H. B. Hildebrand F. Raes J. Sicheritz-Ponten T. Kouskoumvekaki I.. (2013). The chemical interactome space between the human host and the genetically defined gut metabotypes. ISME J. 7, 730–742. 10.1038/ismej.2012.141 23178670 PMC3603391

[B18] Jacobson A. Lam L. Rajendram M. Tamburini F. Honeycutt C. Pham T.. (2018). A gut commensal-produced metabolite mediates colonization resistance to Salmonella infection. Cell Host Microbe 24, 296–307. 10.1016/j.chom.2018.07.002 30057174 PMC6223613

[B19] Jacquet R. LaBauve A. E. Akoolo L. Patel S. Alqarzaee A. A. Lung T. W. F.. (2019). Dual gene expression analysis identifies factors associated with *Staphylococcus aureus* virulence in diabetic mice. Infect. Immun. 87, e00163–e00119. 10.1128/IAI.00163-19 30833333 PMC6479027

[B20] Jamshidi N. Raghunathan A. (2015). Cell scale host-pathogen modeling: another branch in the evolution of constraint-based methods. Front. Microbiol. 6:1032. 10.3389/fmicb.2015.01032 26500611 PMC4594423

[B21] Jenior M. L. Leslie J. L. Young V. B. Schloss P. D. (2017). *Clostridium difficile* colonizes alternative nutrient niches during infection across distinct murine gut microbiomes. mSystems 2, e00063–e00017. 10.1128/mSystems.00063-17 28761936 PMC5527303

[B22] Kiedrowski M. R. Gaston J. R. Kocak B. R. Coburn S. L. Lee S. Pilewski J. M.. (2018). *Staphylococcus aureus* Biofilm growth on cystic fibrosis airway epithelial cells is enhanced during respiratory syncytial virus coinfection. mSphere 3, e00341–e00318. 10.1128/mSphere.00341-18 30111629 PMC6094059

[B23] Kim H. U. Sohn S. B. Lee S. Y. (2012). Metabolic network modeling and simulation for drug targeting and discovery. Biotechnol. J. 7, 330–342. 10.1002/biot.201100159 22125297

[B24] Li P. Xu Z. Sun X. Yin Y. Fan Y. Zhao J.. (2017). Transcript profiling of the immunological interactions between *Actinobacillus pleuropneumoniae* serotype 7 and the host by dual RNA-seq. BMC Microbiol. 17:193. 10.1186/s12866-017-1105-4 28899359 PMC5596872

[B25] Machado D. Andrejev S. Tramontano M. Patil K. R. (2018). Fast automated reconstruction of genome-scale metabolic models for microbial species and communities. Nucleic Acids Res. 46, 7542–7553. 10.1093/nar/gky537 30192979 PMC6125623

[B26] Magnúsdóttir S. Heinken A. Kutt L. Ravcheev D. A. Bauer E. Noronha A.. (2017). Generation of genome-scale metabolic reconstructions for 773 members of the human gut microbiota. Nat. Biotechnol. 35, 81–89. 10.1038/nbt.3703 27893703

[B27] Mazumder M. Gourinath S. (2016). Structure-based design of inhibitors of the crucial cysteine biosynthetic pathway enzyme O-acetyl serine sulfhydrylase. Curr. Top. Med. Chem. 16, 948–959. 10.2174/1568026615666150825142422 26303427

[B28] McHan F. Shotts E. B. (1993). Effect of short-chain fatty acids on the growth of *Salmonella typhimurium* in an *in vitro* system. Avian Dis. 37, 396–398. 10.2307/1591664 8363504

[B29] Minhas V. Aprianto R. McAllister L. J. Wang H. David S. C. McLean K. T. (2019). *In vivo* dual RNA-seq analysis reveals the basis for differential tissue tropism of clinical isolates of *Streptococcus pneumoniae*. bioRxiv 862755 10.1101/862755

[B30] Muñoz J. F. Delorey T. Ford C. B. Li B. Y. Thompson D. A. Rao R. P.. (2019). Coordinated host-pathogen transcriptional dynamics revealed using sorted subpopulations and single macrophages infected with *Candida albicans*. Nat. Commun. 10:1607. 10.1038/s41467-019-09599-8 30962448 PMC6453965

[B31] Niemiec M. J. Grumaz C. Ermert D. Desel C. Shankar M. Lopes J. P.. (2017). Dual transcriptome of the immediate neutrophil and *Candida albicans* interplay. BMC Genomics 18:696. 10.1186/s12864-017-4097-4 28874114 PMC5585943

[B32] Nuss A. M. Beckstette M. Pimenova M. Schmühl C. Opitz W. Pisano F.. (2017). Tissue dual RNA-seq allows fast discovery of infection-specific functions and riboregulators shaping host–pathogen transcriptomes. Proc. Nat. Acad. Sci. U.S.A. 114, E791–E800. 10.1073/pnas.1613405114 28096329 PMC5293080

[B33] Olson W. J. Stevenson D. Amador-Noguez D. Knoll L. J. (2018). Dual metabolomic profiling uncovers Toxoplasma manipulation of the host metabolome and the discovery of a novel parasite metabolic capability. bioRxiv 463075 10.1101/463075 PMC7164669 32255806

[B34] Petrucelli M. F. Peronni K. Sanches P. R. Komoto T. T. Matsuda J. B. da Silva W. A.. (2018). Dual RNA-Seq analysis of *Trichophyton rubrum* and HaCat Keratinocyte co-culture highlights important genes for fungal-host interaction. Genes 9:362. 10.3390/genes9070362 30029541 PMC6070946

[B35] Pittman K. J. Aliota M. T. Knoll L. J. (2014). Dual transcriptional profiling of mice and *Toxoplasma gondii* during acute and chronic infection. BMC Genomics 15:806. 10.1186/1471-2164-15-806 25240600 PMC4177681

[B36] Raghunathan A. Jamshidi N. (2018). Integrated host-pathogen metabolic reconstructions in Metabolic Network Reconstruction and Modeling, ed Fondi M. (New York, NY: Humana Press), 197–217. 10.1007/978-1-4939-7528-0_9 29222755

[B37] Rienksma R. A. Schaap P. J. dos Santos V. A. M. Suarez-Diez M. (2019). Modeling host-pathogen interaction to elucidate the metabolic drug response of intracellular *Mycobacterium tuberculosis*. Front. Cell. Infect. Microbiol. 9:144. 10.3389/fcimb.2019.00144 31139575 PMC6519342

[B38] Sewankambo N. Gray R. H. Wawer M. J. Paxton L. McNaim D. Wabwire-Mangen F.. (1997). HIV-1 infection associated with abnormal vaginal flora morphology and bacterial vaginosis. Lancet 350, 546–550. 10.1016/S0140-6736(97)01063-5 9284776

[B39] Tucey T. M. Verma J. Harrison P. F. Snelgrove S. L. Lo T. L. Scherer A. K.. (2018). Glucose homeostasis is important for immune cell viability during Candida challenge and host survival of systemic fungal infection. Cell Metab. 27, 988–1006. 10.1016/j.cmet.2018.03.019 29719235 PMC6709535

[B40] Uddin R. Tariq S. S. Azam S. S. Wadood A. Moin S. T. (2017). Identification of histone deacetylase (HDAC) as a drug target against MRSA via interolog method of protein-protein interaction prediction. Eur. J. Pharm. Sci. 106, 198–211. 10.1016/j.ejps.2017.06.003 28591562

[B41] Vayssier-Taussat M. Albina E. Citti C. Cosson J. F. Jacques M. A. Lebrun M. H.. (2014). Shifting the paradigm from pathogens to pathobiome: new concepts in the light of meta-omics. Front. Cell. Infect. Microbiol. 4:29. 10.3389/fcimb.2014.00029 24634890 PMC3942874

[B42] Westermann A. J. Barquist L. Vogel J. (2017). Resolving host–pathogen interactions by dual RNA-seq. PLoS Pathog. 13:e1006033. 10.1371/journal.ppat.1006033 28207848 PMC5313147

[B43] Zimmermann M. Kogadeeva M. Gengenbacher M. McEwen G. Mollenkopf H. J. Zamboni N.. (2017). Integration of metabolomics and transcriptomics reveals a complex diet of *Mycobacterium tuberculosis* during early macrophage infection. mSystems 2, e00057–e00017. 10.1128/mSystems.00057-17 28845460 PMC5566787

